# Valorization of Bioactive Compounds from Two Underutilized Wild Fruits by Microencapsulation in Order to Formulate Value-Added Food Products

**DOI:** 10.3390/plants12020267

**Published:** 2023-01-06

**Authors:** Mamadou Abdoulaye Konaré, Nina Nicoleta Condurache, Issiaka Togola, Bogdan Păcularu-Burada, Nouhoum Diarra, Nicoleta Stănciuc, Gabriela Râpeanu

**Affiliations:** 1 Laboratory of Plant and Food Biochemistry, Faculty of Sciences and Techniques (FST), University of Sciences, Techniques and Technologies of Bamako (USTTB), Bamako, Mali; 2 Integrated Center for Research, Expertise and Technological Transfer, Faculty of Food Science and Engineering, Dunărea de Jos University of Galati, 800201 Galati, Romania

**Keywords:** *Balanites aegyptiaca*, *Ziziphus mauritiana*, microencapsulation, biocompounds

## Abstract

Currently, microencapsulation has become a viable method of nutrient delivery for the food industry. This work microencapsulated the bioactive compounds extracted from two neglected species (*Balanites aegyptiaca* and *Ziziphus mauritiana*) by freeze-drying. A combination of wall materials (whey protein and pectin; soy protein and maltodextrin) was chosen to prepare the microcapsules. The phytochemical and physicochemical characterization of the microcapsules was then carried out. The encapsulation yield ranged from 82.77% to 96.05% for *Balanites* and *Ziziphus*, respectively, whereas the efficiency was 76.18 ± 1.39% and 80.93 ± 1.71%. The stimulated *in vitro* gastrointestinal test showed that encapsulation increased the bioavailability of the bioactive compounds. The total carotenoids were the most bioavailable compounds with 85.89 ± 0.06% for *Ziziphus* and 70.46 ± 1.10% for *Balanites*, followed by total flavonoids for *Zizyphus* with 63.27 ± 1.56%. Furthermore, regardless of species or wavelengths, the HPLC analysis resulted in the identification of 17 bioactive metabolites. The predominant one was epicatechin, whose level ranged from 231.52 ± 5.06 to 250.99 ± 3.72 mg/100 g DW in *Ziziphus* and 91.80 ± 3.85 to 116.40 ± 4.09 mg/100 g DW in *Balanites*. In estimating the enzyme inhibition and antioxidant power, both studied fruits showed antidiabetic, inflammatory, and antioxidant effects. These findings suggest that natural bioactive compounds are abundant in the fruits of *Z. mauritiana* and *B. aegyptiaca* and could be a valuable source for the food and pharmaceutical industries.

## 1. Introduction

The management of malnutrition and undernourishment in many African countries continues to be a basic goal of development [1]. The Food and Agriculture Organization (FAO) reports that more than 820 million people worldwide experienced hunger in 2018 (FAO, 2020), with 239 million undernourished persons in Sub-Saharan Africa (SSA) and incidence rates of 22.8%. According to the most current Food Insecurity Experience Scale, more than 700 million people worldwide, or 9.2% of the population, suffered extreme food insecurity in 2018, with Africa having the highest prevalence at 27% (FIES) (FAO, 2020).

Mali especially is facing this scourge. This West African nation’s report showed that 10% of severely stunted children and 27% of chronically malnourished children are under the age of five. Nearly a tenth of children under five years old (9%) are acutely malnourished, i.e., emaciated [2]. This situation is a paradox given the richness of Malian flora and the very interesting wild food plants as demonstrated by many authors [3,4,5]. However, most of these wild food species, which could help to fight malnutrition and food insecurity, are underutilized and even neglected. Among these species, *Ziziphus mauritiana* Lam. and *Balanites aegyptiaca* Del., very frequently available species in Mali, occupy an important place.

*B. aegyptiaca* is a thorny shrub or tree that can reach 10 m in height and is widely distributed in the arid zones of Africa and South Asia [6]. In Mali, the species is mainly found in the Sahelian and Sudanian zone. The leaves and fruits are used in human food during the dry season and the lean periods [5]. As for *Z. mauritiana*, it generally presents as a shrub 4 to 5 m in height, though sometimes reaching a dozen meters. It is a species found throughout semi-arid Africa. It is also found around the Mediterranean in many parts of Asia, Australia, the Caribbean, and tropical America [7]. The fruits of both are consumed fresh, dry, or macerated to produce a drink (juice); they are also sometimes transformed into a dried paste. In addition, these fruits are used by the local population in the treatment of many diseases such as diabetes and hypertension [5,8].

In a recent study, we showed the nutritional and functional values of the fruits of these two species [9] as linked to their high content of nutrients and bioactive compounds such as proteins, polyunsaturated fatty acids, sterols, polysaccharides, vitamins, minerals, phenolic compounds, volatile compounds, and carotenoids [8,10]. It is known that these bioactive compounds play vital roles in human nutrition and the human health system due to their capacity for scavenging oxidative agents, stimulating the immune system, affecting hormone metabolism, and antibacterial and antiviral effects [1,11,12].

However, these chemicals must be accessible to human cells via the digestive system in order for them to fully carry out the aforementioned functions. Among several techniques developed to increase their bio-accessibility, microencapsulation has shown promise in many cases. Microencapsulation consists of entrapping the bioactive molecules within a wall material to protect them against adverse gastrointestinal conditions until they reach the target tissue [13,14,15]. The use of freeze-drying technology for encapsulation in the food industry also enables the large-scale production and development of dry powder as a final product with an ease of storage [16].

To better valorize the underutilized fruits of *Ziziphus mauritiana* and *Balanites aegyptiaca*, this technique could be useful. Hence, the present work is intended to evaluate the phytochemicals, the antidiabetic and anti-inflammatory effects, and the antioxidant activity of the extracts and, thereafter, to microencapsulate the extracts using the freeze-drying technique. After preliminary tests (data not shown), whey proteins and pectin were chosen as the wall material for *Balanites aegyptiaca* extract and soy proteins and maltodextrin for *Ziziphus mauritiana* extract. Furthermore, the bioaccessibility of the biocompounds was evaluated through *in vitro* gastrointestinal digestibility tests of crude and microencapsulated powders from these two fruits. In addition, the microencapsulation was evaluated physicochemical (pH, efficiency, solubility, etc.) and phytochemical (polyphenols, flavonoids, carotenoids, and antioxidant power) parameters. Finally, a scanning electron microscope was used to determine the microcapsule morphologies.

## 2. Results

### 2.1. Preliminary Characterization

The contents of the bioactive compounds and the radical scavenging of DPPH and ABTS are summarized in Table 1 below.

The results shown in Table 1 suggest the richness of bioactive compounds in *Balanites aegyptiaca* and *Ziziphus mauritiana* fruits, and that 70% ethanol is the best solvent to extract the bioactive compounds from these fruits. With this solvent, the highest values were recovered in *B. aegyptiaca*: 14.03 ± 0.44 mg GAE/g DM for total polyphenols, 19.71 ± 0.07 µg β-carotene/g DM for total carotenoids, 16.97 ± 0.12 µg/g DM for β-carotene, and 9.36 ± 0.03 µg/g DM for lycopene. These results confirm the richness of biocompounds in these fruits. In recent work, we obtained similar results with 80% methanol extracts. The level of polyphenols was 1.44 ± 0.08 mg GAE/100 mg for *Balanites aegyptiaca* and 1.72 ± 0.07 mg GAE/100 mg for *Ziziphus mauritiana,* whereas the flavonoid contents ranged from 0.16 to 0.23 mg CE/100 mg for *Balanites* and *Ziziphus*, respectively [10]. The statistical analysis using the Tukey test revealed the highest amounts of bioactive compounds were recorded from the extraction with Ethanol-Water (70:30; *v/v*). Accordingly, the fruit extracts from this solvent (Ethanol-Water) were chosen for further microencapsulation. These findings also agree with those reported by Yahia et al. [11] regarding the methanolic extracts of *Ziziphus* fruits: TPC: 148.75 ± 5.35 mg GAE/100 g DW and TFC: 39.33 ± 3.73 mg QE/100 g DW. Interesting antioxidant power was found in these fruits as seen in the values presented in Table 1. Similar data were also mentioned by Yahia et al. [11] who obtained a total antioxidant capacity of 21.76 ± 0.44 mg GAE/g DW. A lower IC_50_ (14.07 ± 0.46 µg/mL) is an indicator of high antioxidant power.

### 2.2. Antidiabetic and Anti-Inflammatory Activity

The inhibitory activities of one pro-inflammatory enzyme (lipoxigenase) and two enzymes related to metabolic syndrome (α-amylase and α-glucosidase) were analyzed and the data are summarized in Table 2. The relationship between the enzyme’s inhibition and bioactive compounds in addition to antioxidant power was found by searching R^2^ (data shown in Table 3).

Diabetes is a chronic disease and metabolic disorder that presents with a blood glucose level higher than 1 g/L [17] and has become a serious threat to global health that respects neither socioeconomic status nor national boundaries. If not treated properly, it eventually causes major damage to the heart, blood vessels, eyes, kidneys, or nerves. The International Diabetes Federation (IDF) has estimated that 463 million people worldwide have diabetes, the majority living in low-and middle-income countries, with 1.5 million deaths directly attributed to the disease each year [18]. Certain enzymes such as α-amylase and α-glucosidase are highly involved in the regulation of blood sugar levels. They are known as vital enzymes that contribute to the treatment of diabetes by inhibiting carbohydrolases [17]. By inhibiting or limiting their effects, these extracts could contribute to regularizing blood sugar [14,17]. Table 2 shows the efficiency of the extracts to inhibit the effect of three metabolic syndrome-related enzymes. The two fruit extracts presented the same inhibitory effects (*p*-value > 0.05) for these three enzymes. Moreover, the highest effects were recorded against α-glucosidase (more than 95%), followed by α-amylase (from 91.77% to 92.56%); a lesser inhibition rate was registered with lipoxidase, from 16.32 ± 0.99% to 17.98 ± 1.07%. These values are considered highly potent based on the classification in the literature [19].

Therefore, these fruits could be promising to improve diabetes prevention and management to reach the WHO 2025 target of halting the rise in diabetes. The data obtained from the established correlations (Table 3) reveal a high involvement of TPC, TFC, and TC in enzyme inhibitory effects (R^2^ > 0.50). However, α-amylase was the most correlated with these bioactive compounds, with the highest Pearson coefficients for TC (R^2^ = 0.97), TPC, and TFC (R^2^ = 0.94). Moreover, the antioxidant power (DPPH and ABTS) was strongly related to these fruit species’ activity against these enzymes (R^2^ > 0.70) (Table 3). The relationship between various anti-proteinase activities and antioxidant activity also showed a substantial positive correlation of 0.90 [20].

The results obtained from the present work are in agreement with the data from others studies which show that local plants could improve the regulation of the molecular mechanisms involved in diabetes control due to their bioactive compounds [14,21]. Polyphenols and flavonoids are especially evident in antioxidant, anti-inflammatory, antimicrobial, antihypertension, and antidiabetic processes in the human body [22,23,24]. These findings support the results of our recent survey, which showed that the local population in Mali uses the fruits of these two species for the traditional treatment of hypertension [5].

### 2.3. Microencapsulation Parameters

#### 2.3.1. Physicochemical Parameters

The encapsulation yield (EY) and efficiency (EE), the microcapsules’ moisture, and pH are presented in Table 4.

The EY varied from 84.61% for *B. aegyptiaca* to 96.05% for *Z. mauritiana,* whereas the EE ranged from 76.18 ± 1.39% to 80.93 ± 1.71% in the same order. These data are considered satisfying rates in comparison to the mean values from the literature. They corroborate the conclusions of Tao et al. [25] who suggested that using mixtures of coating agents in different combinations might be more efficient than the individual compounds alone. Using different combinations of WPI and maltodextrin (MDX), Karrar et al. [26] recorded EY values ranging from 85.25 to 92.80% and recorded an EE of 90.78 ± 0.80% when combining WPI with maltodextrin in a 3:1 ratio. Li et al. [27] succeeded in microencapsulating 76.4–87.4% of polyphenolics from ethanolic plum extract by spray drying. Moreover, with a combination of maltodextrin, gum arabic, and gelatin to microencapsulate anthocyanins from barberry (*Berberis vulgaris*) extract by spray drying, Akhavan Mahdavi et al. [28] recorded encapsulation efficiencies from 89.06% to 96.21%. The work conducted by da Rosa et al. [16] obtained similar EE results, from 82.53 to 85.79%, while working with one material (MDX) at different ratios and temperatures. The highest encapsulation yield of *Ziziphus* could be related to the properties of maltodextrin because this compound presents a high solubility in water, low viscosity, and low sugar content [16]. Nevertheless, these values varied from 20 ± 2 to 93 ± 2% for Dumitrascu et al. [29] by encapsulating anthocyanins using SPI/WPI at different treatments. They concluded that both the coating material and the drying technique affected encapsulation efficiency. It is seen in the literature that the encapsulation yield is affected by many factors such as the dry matter of the extract, wall material type and composition, and parameters of the encapsulated powder [6,29,30]. On the other hand, the higher carbohydrate contents of these fruits could negatively influence the yield during freezing procedures [30].

The determination of pH and humidity is important given that these parameters are involved in storage stability. The higher levels of these parameters the powder possesses, the more microbial growth and enzymatic reactions are accelerated [29]. The moisture obtained from the microcapsule powders ranged from 5.97 ± 0.65% to 7.34 ± 0.32%. Similar results were recorded by da Rosa et al. [16] with 4.77 to 7.00% using WPI at different percentages. Dumitrascu et al. [26] also reported similar results varying from 5 and 10% with WPI at different temperatures. Moreover, other studies reported a lesser moisture content (<4%) which is considered a desirable characteristic for microcapsule powders [26]. Given that various parameters may affect the moisture content, the deviations from the values found in the present study may be attributed to the wall material (type and composition) and the characteristics of the samples [31].

Solubility is the final particle dissolution stage, which is also a key factor in food quality and is analyzed by testing the dissolution rate of the microparticle in water. In this study, the solubility ranged from 82.13 to 83.50% (Table 4). This is consistent with the results obtained by Karrar et al. [26] which were from 86 to 93.84%. These values could confer the good storage stability of the powders and, consequently, ease preservation in addition to enabling efficient processing in the food industry. The poorer the solubility of the powder, the more difficult the processing, resulting in possible economic losses [16,26].

#### 2.3.2. Chemical Parameters and Antioxidant Power

Table 5 summarizes the chemical parameters and antioxidant power of microencapsulated powders.

These recorded data from the microcapsules show that the encapsulation technique improves the phytochemical contents of the extracts. The amounts of TPC (from 20.75 ± 0.86 to 23.09 ± 1.05 mg GAE/g DM) and TFC (from 13.22 ± 0.19 to 15.91 ± 1.38 mg QE/g DM) increased greatly with microencapsulation. This may be because pectin and maltodextrin can bind to these specific compounds. These data are in line with those of Azarpazhooh et al. [32] who registered significantly higher contents of anthocyanins (from 59.7 to 73.1 mg GA/kg) by increasing the percentage of maltodextrin from 5 to 15%. The literature showed the involvement of a number of bioactive compounds, especially flavonoids and polyphenols, in DPPH and ABTS radical scavenging from plant extracts and food [24,32]. Moreover, the highest antioxidant effect was obtained from ABTS radical scavenging for *Z. mauritiana* with 86.81 ± 1.47 µM TE/g and from DPPH radical scavenging for *B. aegyptiaca* with 17.87 ± 1.77 µM TE/g. These differences between the extracts could be due to the reaction mechanisms and the type or structure of the biocompounds involved in each method. A strong relation (R^2^ > 0.70 in most cases) was found between the antioxidant power and the bioactive compounds. It is reported in the literature that the TFC is slightly correlated with DPPH radical scavenging (R^2^ = 0.473) but highly correlated with total antioxidant capacity (R^2^ = 0.85) [11].

#### 2.3.3. Stability of Microencapsulated Compounds under Storage

**Figure 1:**
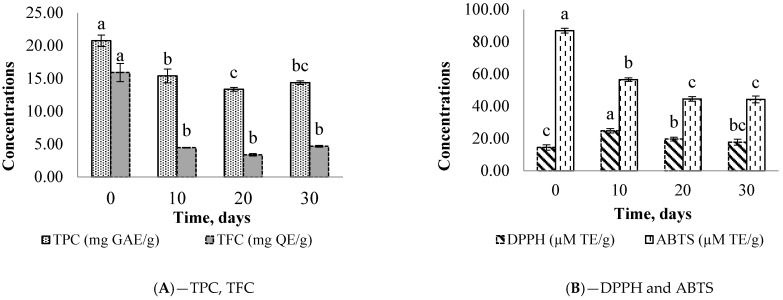
Storage stability of TPC, TFC (**A**), DPPH, and ABTS (**B**) from the microencapsulated extracts of *Ziziphus mauritiana.* Different letters (a–c) for the same parameter show a significant difference between the means at 0.05.

**Figure 2:**
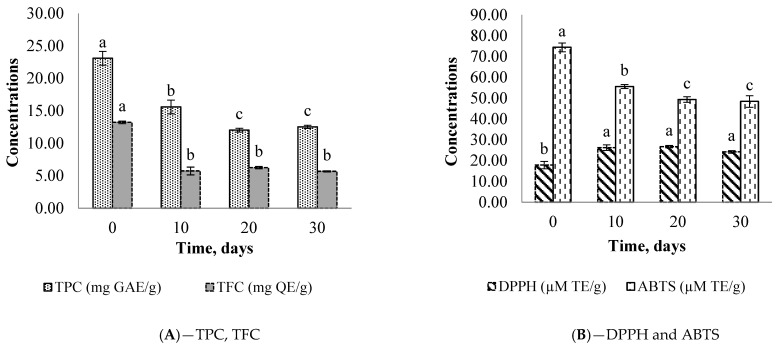
Storage stability of TPC, TFC (**A**), DPPH, and ABTS (**B**) from the microencapsulated powder of *Balanites aegyptiaca.* Different letters (a–c) for the same parameter show a significant difference between the means at 0.05.

The results of a 30-day study on the stability of the microencapsulated compounds are shown in Figure 1 and Figure 2.

It appears that the decrease in TFC and TPC is associated with ABTS scavenging radicals between 0 and 10 days for both microcapsules. This behavior is confirmed by Pearson’s positive correlation (R^2^ > 0.84). However, it is interesting to note the upsurge in DPPH radical scavenging at the same period; this could be due to the involvement of other molecules (carbohydrates, proteins, or lipids) in the reaction mechanism of the DPPH radical. These decreases in TFC and TPC could also be attributed to the microcapsule moisture contents which are higher (from 5.97 ± 0.65% to 7.34 ± 0.32%) than the expected value from the literature (<4%) for better storage [26]. In contrast, good protection was observed with carotenoid compounds (TC, BC, and LC). From T_0_ to T_30_, the TC values changed from 2.46 to 2.67 µg/g and 11.11 ± 0.02 to 10.98 ± 0.05 µg/g, respectively, for *Ziziphus* and *Balanites* extracts.

#### 2.3.4. SEM Analysis

**Figure 3:**
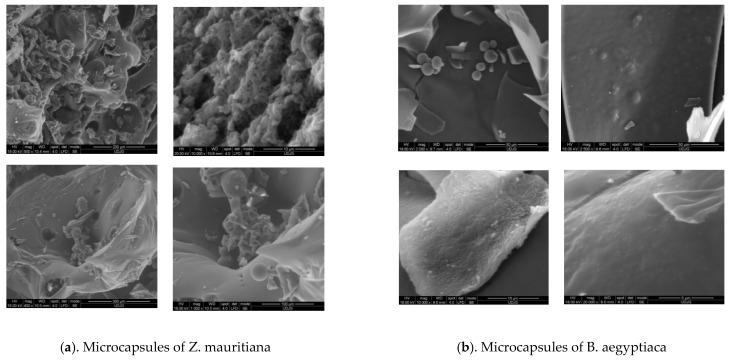
Structure of the powders obtained by *Ziziphus mauritiana* (**a**) and *Balanites aegyptiaca* (**b**) extracts microencapsulation.

The SEM images presented in Figure 3 show the distribution of particles from the powders. The irregular structures of the microcapsules appear as flakes and amorphous glass-like structures. Indeed, these structures are well known as a good indicator of the protection of the entrapped molecules against high temperatures and air oxygen [32]. Many similar data have been obtained in the literature.

An amorphous glass-like structure was visible under the SEM, which is expected to protect the molecules trapped inside from heat and oxygen exposure. Similar images, characteristic of freeze-dried components encapsulated in MDX pigments, are reported by Azarpazhooh et al. [32]. Shrinkages were observed for these samples which, according to Le Priol et al. [33], is typical of microparticles produced with proteins as wall materials. Gong et al. [34] suggested that this phenomenon might be due to the rapid formation of a dried crust layer on the surface of the microparticles followed by the high flux of moisture leaving the particle during drying.

### 2.4. In Vitro Stimulated Gastrointestinal Digestibility

**Figure 4:**
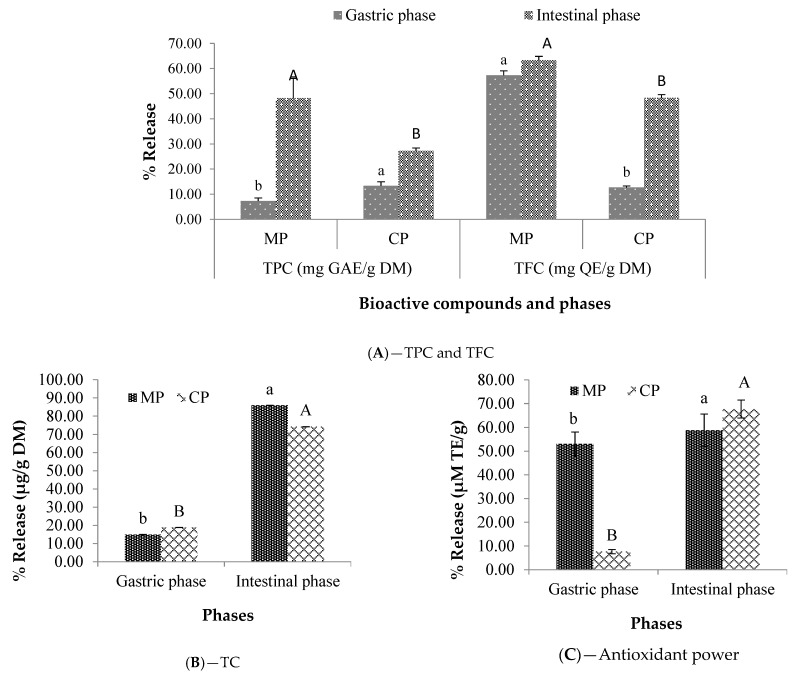
TPC, TFC (**A**), TC (**B**), and antioxidant activity (**C**) release (%) after 2 h of *in vitro* gastric and intestinal digestion of *Ziziphus mauritiana* crude and microencapsulated extracts. MP: Microencapsulated powder; CP: Crude powder; TPC: Total polyphenolic contents; TFC: Total flavonoid contents; TC: Total carotenoids. For each biocompound, the gastric phase averages that do not share any lower-case letters are significantly different at the 0.05 threshold; the same applies to the averages of the intestinal phase that do not share any upper-case letters.

**Figure 5:**
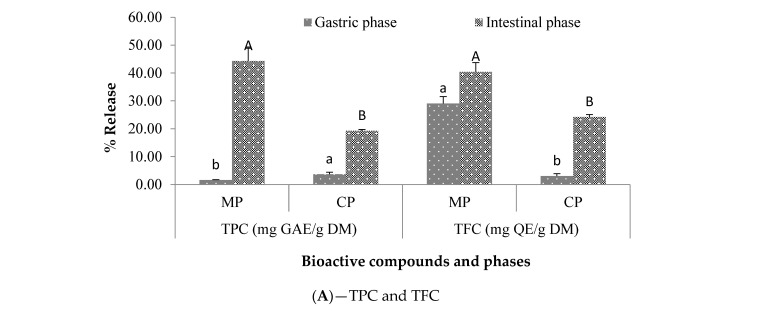

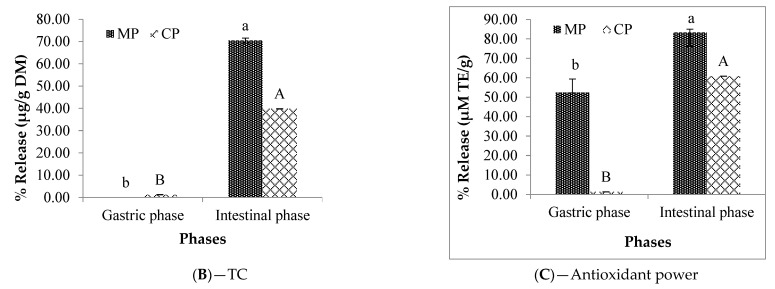
TPC, TFC (**A**), TC (**B**), and antioxidant activity (**C**) release (%) after 2 h of *in vitro* gastric and intestinal digestion of *Balanites aegyptiaca* crude and microencapsulated extracts. MP: Microencapsulated powder; CP: Crude powder; TPC: Total polyphenolic contents; TFC: Total flavonoid contents; TC: Total carotenoids. For each biocompound, the gastric phase averages that do not share any lower-case letters are significantly different at the 0.05 threshold; the same applies to the averages of the intestinal phase that do not share any upper-case letters.

The results of the *in vitro* stimulated gastrointestinal digestibility of encapsulated (MP) and crude powders (CP) are illustrated in Figure 4 and Figure 5. For each powder (encapsulated and non-encapsulated/crude), the highest release percentages of biocompounds were observed during the intestinal step. Moreover, all the biocompounds released more with the microencapsulated powders compared to the crude powders during the intestinal step. The total carotenoids were the most bioavailable compounds with 85.89 ± 0.06% for *Ziziphus* and 70.46 ± 1.10% for *Balanites*, followed by the total flavonoids for *Ziziphus* with 63.27 ± 1.56% and polyphenols for *Balanites* with 48.23 ± 7.84%. The release of carotenoids is influenced by the extraction method and their interference with chlorophyll as mentioned by Biehler et al. [35] who released carotenoids from consumed fruits and vegetables, without saponification, with significantly higher results (from 6.0 ± 0.4 to 4.2 ± 0.5 mg/100 g) due to chlorophyll interference.

As for the antioxidant powder during the intestinal phase, higher values were registered from *Balanites* (83.33 ± 1.73%) microencapsulated powder extracts against 58.84 ± 6.76% for those of *Ziziphus* at the same phase. Thereby, the microencapsulation could efficiently enhance the bioavailability of these compounds in the gastrointestinal route. As noted in the literature, the bioavailability and the bioaccessibility of compounds are limited when ingested from whole crude powder [12] because of the rigid cell membranes which are not or only partially degraded by the digestive enzymes present in the stomach and/or intestine [36]. Many other factors (such as the type of encapsulating agents, the nature of the combination, the structure and bioactive composition of the extract, etc.) have been reported in the literature to influence the release of biocompounds as well [16,27,30]. Certain authors have thought that the main mechanisms involved in the core release are diffusion, degradation, use of solvent, pH, temperature, and pressure [29].

### 2.5. HPLC Analysis of Total Polyphenolic and Flavonoid Compounds

**Figure 6:**
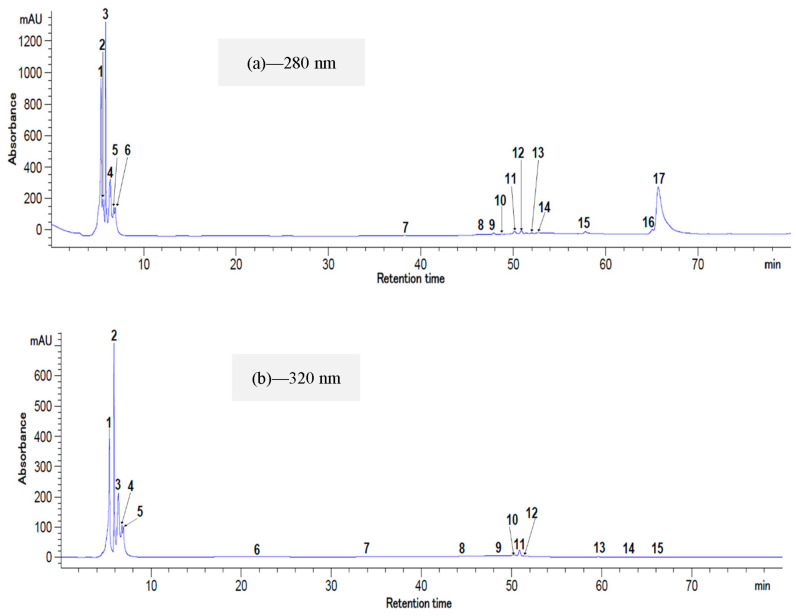
HPLC chromatogram for the flavonoids and polyphenols from *Ziziphus mauritiana* extract at 280 nm (**a**) and 320 nm (**b**). Peak identifications: (**a**) 2—Gallic acid, 3—Chlorogenic acid, 4—(-)—Epicatechin, 5—Caffeic acid, 8—Quercetin, 13—Isorhamnetin, 1, 6, 7, 9–12, and 14–17—unidentified peaks; (**b**) 3—(-)—Epicatechin, 4—Caffeic acid, 6—p-coumaric acid, 9—Quercetin, 1, 2, 5, 7, 8, and 10–15—unidentified peaks.

**Figure 7:**
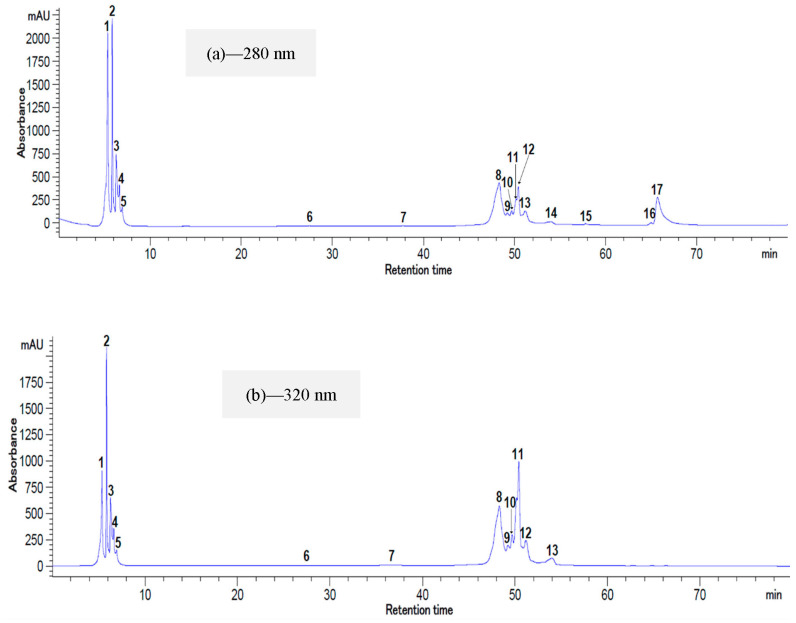
HPLC chromatogram for the flavonoids and polyphenols of *Balanites aegyptiaca* extract at 280 nm (**a**) and 320 nm (**b**). Peak identifications: (**a**) 2—Chlorogenic acid, 3—(-)—Epicatechin, 5—Caffeic acid, 1, 4, and 6–17—unidentified peaks; (**b**) 2—Chlorogenic acid, 3—(-)—Epicatechin, 5—Caffeic acid, 1, 4, and 6–13—unidentified peaks.

The profile of the total polyphenolic and flavonoid compounds from *Ziziphus* (Figure 6a,b) and *Balanites* (Figure 7a,b) extracts was determined using HPLC-DAD analysis. The identification of these compounds was carried out at two wavelengths (280 and 320 nm) based on the retention time and by comparing them to the available standards. The chromatographic profile of the extracts highlighted the presence of 17 compounds at 280 nm for both species. In addition, at 320 nm, 15 compounds were detected in the fruits of *Ziziphus* and 13 for *Balanites*.

Table 6 presents the identified biocompounds and their concentrations. Seven peaks were identified in the fruits of *Ziziphus* against only three peaks for *Balanites*. The (-)—epicatechin was the main flavonoid compound with the highest amount, from 231.52 ± 5.06 to 250.99 ± 3.72 mg/100 g DW for *Ziziphus* and from 91.80 ± 3.85 to 116.40 ± 4.09 mg/100 g DW for *Balanites*. All the determined compounds in this study are already reported in the literature for *Balanites* [37] and *Ziziphus* [11]. However, quercetin, isorhamnetin, and their derivatives have been found in *Balanites* fruits by the same authors. Additionally, their work reported a lower relative content (1.21 ± 0.01 µg/g) of quercetin in *Ziziphus* fruits based on their HPLC analysis [11]. Variations among the concentrations of phenolic acids from *Ziziphus*, such as chlorogenic, caffeic, and p-coumaric acid, were reported by Memon et al. [38], with the results being influenced by the analyzed species or by the extraction protocols. The microencapsulation system used for *Balanites* extract stabilization could explain the lack of quercetin in our HPLC results. On the contrary, quercetin levels between 8.26–24.71 mg/100 g were quantified from the same raw material [39]. The caffeic acid concentration from the *Balanites* microencapsulated powder is comparable with the results reported by Khamis et al. [21] who determined variations between 0.02 and 0.05 mg/100 g DW for fruit samples originating from different geographical areas.

## 3. Materials and Methods

### 3.1. Materials

The plant material used in this study was derived from *Balanites aegyptiaca* and *Ziziphus mauritiana* fruits, harvested at the local market of Banamba in Mali. They were dried at room temperature, and the pulp was recuperated, ground, and stored at room temperature until further analysis.

### 3.2. Methods

#### 3.2.1. Extraction

Ten grams (10 g) of fruit powder were dissolved in 100 mL of 70% ethanol mixed with glacial acetic acid (9:1 ratio) or 70% ethanol only. A digital ultrasonic cleaner (model number Argo Lab DU-32) was used for the extraction which took place for 30 min at a temperature of 25 to 30 °C and Power of 5. The solutions were combined and centrifuged for 20 min at 6000 rpm and 25 °C. Then, the collected supernatants were concentrated under reduced pressure at 40 °C (RVC 2-18 CDplus, Christ, Osterode am Harz, Germany) and stored cold before analysis.

#### 3.2.2. Characterizations

The content characterizations of total phenols (TPC), total flavonoids (TFC), total carotenoids (TC), lycopene, and carotene were performed using the spectrophotometric methods described in the literature.

➢ Total phenolic contents (TPC)

By using the Folin Ciocalteau method, the concentration of phenolic compounds was determined. In addition to 7.9 mL of water, 0.5 mL of the Folin Ciocalteau reagent was added to 100 L of extract. After homogenization and 10 min of rest at room temperature, 1.5 mL of 20% disodium carbonate (Na_2_CO_3_) was added to the reaction mixture. After incubation for 60 min at room temperature in the dark, the absorbances were measured at 765 nm by a UV/VIS spectrophotometer (Biochrom Libra S22, Cambridge, UK). Under the same circumstances, a calibration curve was created using Gallic acid as the standard. The results were given as milligrams of Gallic acid equivalent per gram of dry matter extract (mg GAE/g DM).

➢ Total flavonoids contents (TFC)

Flavonoids were determined using the aluminum trichloride reagent (AlCl_3_) according to the protocol described by Keita et al. [23]. This involved the addition of 500 μL of extract to 2 mL of distilled water and 150 μL of 5% sodium nitrite (NaNO_2_). Next, 150 μL of 10% aluminum trichloride (AlCl_3_) was added to the mixture after 5 min and re-incubated for another 6 min at room temperature. Subsequently, 1 mL of 1 M sodium hydroxide (NaOH) was added. The mixture reaction was homogenized and the absorbances of the pinkish solution were measured at 510 nm using a UV/VIS spectrophotometer (Biochrom Libra S22, Cambridge, UK). The concentrations of the total flavonoids were determined from the calibration curve obtained using quercetin as a standard. The results were expressed in milligrams (mg) of quercetin equivalent per gram of dry matter (mg QE/g DM).

➢ Total carotenoid, β-carotene, and Lycopene contents

The total carotenoid, β-carotene, and lycopene contents were analyzed following a modified version of the method described by Canan et al. [40]. To quantify the levels of these compounds, 2 mL of extract (100 mg/mL) solubilized in the extraction solvent was introduced into the UV quartz cuvette and the absorbance was read at λ = 450 nm, 470 nm, and 503, respectively, for carotenoids, β-carotene, and lycopene. Their concentrations were calculated from Equation (1) below.

(1) $$\mathrm{Contents}\;(\mathrm{mg}/g\;\mathrm{DM})=\frac{\mathrm{Abs}\;\times \;V}{m\;\times \;L\;\times \varepsilon \;}\;\times \;100\;$$

Abs—Absorbance of the sample;

V—Volume of solvent used to solubilize the concentrated extract;

M—Mass/weight of concentrated extract;

L—Length of the optical path of the cuvette (1 cm for quartz cuvette);

Ε—Extinction coefficient, which is 2500 for carotenoids, 2590 for β-carotene, and 3450 for lycopene.

#### 3.2.3. Antioxidant Activity

The antioxidant activities of extracts were measured by DPPH (1.1-diphenyl-2-picrylhydrazyl) and ABTS (2.2 9-azinobis-3-ethylbenzothiazoline 6-sulfonic acid) scavenging methods.

➢ DPPH radical scavenging

The DPPH radical scavenging assay was carried out according to a previously reported method [13]. To 0.1 mL of extract, a volume of 3.9 mL of DPPH stock solution (0.1 M) was added. After a 30 min incubation period at room temperature in the dark, the absorbance at 515 nm was measured against a control solution without extract. The scavenging potency of DPPH was expressed as µmol Trolox equivalents per gram of dried extract (µmol TE/g DM) using a calibration curve.

➢ ABTS radical scavenging

A discoloring assay applicable to both aqueous and lipophilic systems was used to perform the ABTS radical scavenging according to the method of Re et al. [41] and updated by Siswoyo et al. [42]. ABTS^.+^ was generated by mixing 7 mM ABTS and 2.45 mM potassium persulfate after incubation at room temperature (25 °C) in the dark for 16–24 h. ABTS stock solution was prepared, and its absorbance was adjusted at 0.700. Then, 50 µL of the extract was mixed with 1950 µL of adjusted ABTS stock solution. After 2 h at room temperature in the dark, the absorbance at 734 nm was read. The ABTS scavenging percentage was expressed as µmol Trolox equivalents per gram of dried extract (µmol TE/g DM) using a calibration curve.

#### 3.2.4. Antidiabetic Activity

The antidiabetic activity was determined based on the inhibitory activity of two key enzymes involved in metabolic syndrome: α-amylase and α-glucosidase.

➢ α-amylase inhibition activity

To analyze the inhibitory effect against α-amylase, the method described by Cai et al. [17] with minor modifications performed. This method involved mixing 100 µL of 1 mg/mL α-amylase (Sigma Aldrich, Darmstadt, Germany, 10065-10G from *Aspergillus oryzae*) in a phosphate buffer of sodium (PBS) at 0.1 M with a pH of 6.9 with 100 µL of extract at known concentrations. After 5 min of rest at room temperature, the enzyme reaction was started by adding 100 µL of 1% starch solution into the mixture, followed by incubation for 20 min at 37 °C. Afterward, the enzyme reaction was stopped by adding 200 µL of 3.5-Dinitrosalicylic acid solution (DNS). Next, the samples were kept in boiled water at 100 °C for 5 min, followed by volume adjusting to 2.5 mL with distilled water. The absorbances were read at 540 nm with a UV/VIS spectrophotometer (Biochrom Libra S22). The inhibition percentages were determined using Equation (2):

(2) $$\%\;\mathrm{Inhibition}=\frac{\left(\mathrm{Absorbance}\;\mathrm{of}\;\mathrm{blank}\;-\;\mathrm{Absorbance}\;\mathrm{of}\;\mathrm{Sample}\right)}{\mathrm{Absorbance}\;\mathrm{of}\;\mathrm{Sample}}\;\times \;100\;$$

➢ α-glucosidase inhibition activity

The inhibitory effect of α-glucosidase was analyzed following the protocol reported in the literature [43]. Briefly, 100 µL of extract at known concentrations were mixed with 50 µL α-glucosidase at 1 mg/mL (powder from *Saccharomyces cerevisiae*: Sigma Aldrich G5003-100 UN) dissolved in PBS 0.1 M at a pH of 6.9). After 5 min of pre-incubation at room temperature, 1.6 mL of PBS 0.1 M, pH 6.9, and 50 µL of p-nitrophenyl-α-D-glucopyranoside 25 mM (Sigma Aldrich, N1377-5G) were added to the mixture reaction. The mixture was kept for 20 min at 37 °C and supplemented with 800 µL of Na_2_CO_3_ 0.2 M. The inhibitory effect of α-glucosidase, expressed as a percentage (%), was obtained from the absorbance read at 405 nm and the same equation used for α-amylase, Equation (2).

#### 3.2.5. Anti-Inflammatory Activity

The anti-inflammatory performance was estimated by the inhibition of one pro-inflammatory enzyme: lipoxygenase, according to Iqbal et al. [20], with small amendments.

The lipoxygenase inhibition was performed throughout the oxidation of linoleic acid into the corresponding hydroperoxides. The reaction consisted of mixing 50 µL of lipoxidase from soybeans (purchased at Sigma Aldrich L7395-15MU made in the UK) at 1 mg/mL in 0.1 M PBS at a pH of 9.0 and 100 µL of extract at different concentrations. After 5 min rest at room temperature, 50 µL of 50 µM linoleic acid and 1.8 mL of 0.1 M PBS at a pH of 9.0 were added. Thereafter, the reaction mixture was incubated for 20 min at 37 °C and the absorbance was read at 234 nm against a blank without extract using a quartz cuvette. Equation (2) was used to calculate the inhibition performance (%).

#### 3.2.6. Microencapsulation

##### Preparation of Powder Microencapsulation

For microencapsulation, the concentrated extracts obtained from ethanol 70% were used due to their high level of bioactive compounds. A combination of wall materials was applied: whey protein isolate (WPI) and pectin for *Balanites aegyptiaca* extract; soy protein isolate (SPI) and maltodextrin for *Ziziphus mauritiana*.

The method recently described by Karrar et al. [26] was followed. First, a solution of 1% WPI or SPI in 100 mL of ultrapure water was prepared. Subsequently, 1 g of pectin or maltodextrin was added, and the mixture was placed under magnetic stirring (IKA T18 basic, Staufen, Germany) for 3 h at 40 °C and 400 rpm. Thereafter, the fruit extracts were added in order to obtain 30 mg/mL for *Balanites* and 25 mg/mL for *Ziziphus*. The mixture reaction was homogenized at 25 °C and 400 rpm. After 1 h, the pH was checked and adjusted to 4–6 (if applicable). Lastly, the mixture reaction was frozen overnight before submitting for freeze-drying (Christ Alpha 1-4 LD plus, Martin Christ, Osterode am Harz, Germany) at −40 °C under a pressure of 0.10 mBar.

##### Physicochemical Properties of Microencapsulated Powder

➢ Humidity, pH, and solubility:

The pH of microencapsulated powders was analyzed by a pH meter (Mettler Toledo S20K) after dissolving 1 g of powder in 10 mL of distilled water [32].

A moisture analyzer (MF-50, A&D Co Ltd., Tokyo, Japan) was used to test the humidity content of the microcapsule powders using the gravimetric method until it registered a constant weight [32].

As for the solubility of microencapsulated powders, the method recently reported by Karrar et al. [26], with minor modifications, was used. In brief, 1 g of powder was mixed and transferred into 100 mL of distilled water and homogenized by a vortex mixer (Benchmark Scientific, Sayreville, New Jersey, USA). Afterward, the solution was centrifuged at 5000 for 20 min (Mikro 320 R Hettic, Tuttlingen, Germany). The collected supernatant was dried at 105 °C. The solubility of the microcapsules was calculated using the following Equation (3).

(3) $$\mathrm{Solubility}\;(\%)=\frac{\mathrm{Powder}\;\mathrm{of}\;\mathrm{the}\;\mathrm{supernatant}\;\left(g\right)}{\mathrm{Test}\;\mathrm{intake}\;\left(g\right)}\;\times \;100$$

➢ Determination of encapsulation yield (EY)

The microencapsulation yield (Y) of the freeze-dried extracts was estimated using Equation (4) [32].

(4) $$\mathrm{Yield}\;(\%)=\frac{\mathrm{Ws}}{\mathrm{Ww}\;+\;\mathrm{Wt}}.\;\times \;100\;$$

where Ws is the weight of encapsulated powder (g), Ww is the weight of wall materials (g), and Wt is the weight of test intake extract (g).

➢ Determination of encapsulation efficiency (EE)

The method reported by Dumitrascu et al. [29] was used to analyze the microencapsulation efficiency (EE) throughout the measurement of total flavonoids. For the TFC, 0.5 g of microcapsules were dissolved in 10 mL of methanol-glacial acetic acid-water (50:8:42; *v/v/v*) by thoroughly vortexing and then subjecting to ultrasonication (Digital ultrasonic cleaner; Argo Lab DU-32) for 30 min at 40 kHz. As for the surface flavonoid content (SFC), 0.5 g of microcapsules were mixed in 10 mL mixture solvent (methanol-ethanol: 1:1; *v/v*) and vortexed thoroughly for 1 min. Both (TFC and SFC) samples were then centrifuged for 10 min at 14,000 rpm at 4 °C (Mikro 22 R Hettich, Tuttlingen, Germany). Flavonoid contents in the collected supernatants were evaluated as described above using an aluminum trichloride reagent [23] and expressed as milligrams of quercetin equivalents per dry extract (mg QE/g DM).

The TPC, the antioxidant scavenging radical DPPH, and ABTS were also analyzed in the encapsulated powder following the same methods previously described.

The efficiency (EE) was calculated from Equation (5):

(5) $$\mathrm{EE}\;(\%)=\frac{\mathrm{TFC}\;-\;\mathrm{SFC}}{\mathrm{TFC}}\;\times \;100$$

where EE is the encapsulation efficiency and SFC is the surface flavonoids content.

➢ Stability of microencapsulated compounds under storage

For the analysis of storage stability, da Rosa et al.’s [16] methodology was used. For this, the microcapsules were sealed and then stored at 4 °C in Falcon tubes with light protection. The samples were examined every 10 days for 30 days in order to assess the TFC and TPC, TC, and antioxidant power.

➢ Scanning Electron Microscopy (SEM)

A scanning electron microscope was used to examine the powder morphologies [44]. Samples were placed on carbon foil and coated with 5 nm gold thickness in an argon atmosphere. The surface micrographs of samples were obtained by exposition to accelerated electron beams of 20 kV, a pressure of 0.6 mmHg, and a spot size of 4 using an FEI Quanta 200 (SPI Spattering, West Chester, PA, USA). Lastly, the pictures of the microcapsules were taken at magnifications of 20,000× and 500,000× [44].

#### 3.2.7. *In Vitro* Digestibility

To investigate *in vitro* digestion, a protocol from the literature was used [29]. The simulated gastric fluid (SGF) was prepared with pepsin from porcine gastric mucosa (Sigma Aldrich, USA) at 1 mg/mL in 0.1 M HCl at a pH of 2.0. Almost 1 g of powder was dissolved in 5 mL 10 mM Tris-HCl buffer solution of pH 7.7. After homogenization, simulated gastric fluid (SGF) in a 1:10 ratio, constituted of pepsin from porcine gastric mucosa (Sigma Aldrich, USA) at 1 mg/mL in 0.1 M HCl at a pH of 2.0, was added to the mixture. The mixture reaction was incubated for 2 h, at 37 °C, 150 rpm on an SI-300R orbital shaking incubator (Medline Scientific, Chalgrove, UK). At 0, 60, and 120 min, 2 mL were taken and centrifuged at 14,000 rpm for 10 min at 4 °C for immediate characterization (TPC, TFC, TC, and antioxidant activity).

After 120 min of the gastric step, a volume of 5 mL was mixed with simulated intestinal fluid (SIF) (1:2 ratio) based on porcine pancreatin at 2 mg/mL in 0.9 M sodium bicarbonate at pH 7.0. The mixture reaction was re-incubated for 2 h, at 37 °C, 150 rpm on an SI-300R orbital shaking incubator. At 0, 60, and 120 min, the phytochemical analysis (TPC, TFC, TC, and antioxidant activity) was done on the supernatant taken after centrifuging at 14,000 rpm for 10 min at 4 °C.

For each sample, the percentage of bioactive compounds released after 2 h of incubation with SGF and SIF was obtained using the following Equation.

(6) $$\%\;\mathrm{Release}=[1\;-\;\frac{\mathrm{Ci}}{\mathrm{Cf}}]\;\times \;100$$

where Ci is the initial concentration of phytochemicals and Cf is the final concentration of phytochemicals.

#### 3.2.8. Determination of Flavonoids and Polyphenolic Compounds by HPLC

The separation and identification of the bioactive compounds from the fruits of *Balanites aegyptiaca* and *Ziziphus mauritiana* powders were carried out by an Agilent 1200 HPLC system equipped with an autosampler, degasser, quaternary pump system, multi-wavelength detector (MWD) and column thermostat (Agilent Technologies, Santa Clara, CA, USA). A Synergi Max-RP-80 Å column (250 × 4.6 mm, 4 µm particle size, Phenomenex, Torrance, CA, USA) protected by a guard column (Phenomenex, Torrance, CA, USA) was used for the bioactive compounds’ separation. Specifically, for the separation of the flavonoids and polyphenolic compounds, solvent A, made of ultrapure water: acetonitrile: formic acid at an 87:3:10 ratio, and solvent B, made of ultrapure water: acetonitrile: formic acid at 40:50:10 ratio, were used [45], respectively, with minor modifications. These solvents were flushed into the system with a flow rate of 0.50 mL/min at 30 °C using an injection volume of 20 µL and the following gradient: 0 min—94% A; 20 min—80% A; 35 min—60% A, 40 min—40% A, and 45 min—10% A. The method runtime was 80 min, and the compounds of interest were detected at 280 nm and 320 nm.

Prior to the HPLC separation, the bioactive compounds from a sample aliquot (1 g) were extracted with a mixture of methanol 80% (*v/v*) and formic acid (9:1) at room temperature for 12–16 h at 150 rpm [46].

The identification of the bioactive compounds from the analyzed samples was made by comparing the retention times and peak areas with those obtained for standard solutions of bioactives. The identified compounds were quantified by external calibration curves using the peak area. Data acquisition was made by Chemstation software, version B.04.03 (Agilent Technologies, Santa Clara, CA, USA). Results were expressed in mg/100 g DW powder.

### 3.3. Statistical Analysis

The registered data are presented as the mean ± standard deviation (SD) of triplicate analyses. The correlation between some bioactive compound levels found in the extracts and the biological activities was analyzed by the Pearson test correlation coefficients with 95% confidence. For the statistical analysis, one-way ANOVA was preceded by checking the normality followed by the Tukey test (*p* < 0.05) to compare the means using Minitab 18.1.

## 4. Conclusions

This work was undertaken in order to contribute to the valorization of bioactive compounds from the fruits of *Balanites aegyptiaca* and *Ziziphus mauritiana* by microencapsulation in order to formulate value-added food products. Firstly, after a preliminary test, the enzyme inhibition and antioxidant power of the extracts were assessed. The extracts were encapsulated using a combination of wall materials. The obtained results showed that these species were rich in antioxidants and possessed good antidiabetic and anti-inflammatory properties. The stimulated *in vitro* gastrointestinal test showed that the encapsulation technique increased the bioavailability of bioactive compounds. Moreover, the HPLC analysis confirmed the richness of these species in bioactive compounds. Based on these findings, the fruits of *Z. mauritiana* and *B. aegyptiaca* deserve to be more valorized to contribute to the fight against malnutrition and improve the health of the local inhabitants of Mali. In addition, the obtained microcapsules may be successful candidates for natural and antioxidant pigments to replace the synthetic ones frequently used in the food industry, thus contributing to the development of new value-added food products.

## Figures and Tables

**Table 1:** Bioactive compound levels extracted by UAE from *Ziziphus mauritiana* and *Balanites aegyptiaca fruits*.

Parameters	*Z.* *mauritiana*		*B.* *aegyptiaca*	
	EtOH 70%-AA	EtOH 70%	EtOH 70%-AA	EtOH 70%
Total polyphenols (mg GAE/g DM)	13.40 ± 0.33 ^a^	13.99 ± 0.50 ^a^	10.17 ± 0.42 ^b^	14.03 ± 0.44 ^a^
Total flavonoids (mg EQ/g DM)	7.32 ± 0.26 ^a^	3.92 ± 0.23 ^b^	6.73 ± 0.16 ^a^	5.63 ± 0.12 ^b^
Total carotenoids (µg/g DM)	3.28 ± 0.01 ^b^	5.14 ± 0.00 ^a^	11.59 ± 0.00 ^b^	19.71 ± 0.07 ^a^
β-Carotene (µg/g DM)	2.38 ± 0.00 ^b^	4.17 ± 0.01 ^a^	8.52 ± 0.00 ^b^	16.97 ± 0.12 ^a^
Lycopene (µg/g DM)	1.18 ± 0.00 ^b^	2.50 ± 0.01 ^a^	3.99 ± 0.00 ^b^	9.36 ± 0.03 ^a^
DPPH (µM TE/g DM)	21.45 ± 0.08 ^b^	23.79 ± 0.80 ^a^	19.76 ± 0.16 ^b^	20.75 ± 0.28 ^a^
ABTS (µM TE/g DM)	21.35 ± 0.36 ^a^	21.84 ± 0.55 ^a^	21.18 ± 0.40 ^a^	16.86 ± 0.428 ^b^

Different letters (a–b) for the same parameter and the same plant species show a significant difference between the means at 0.05. EtOH 70%-AA: Ethanol-Acetic acid-Water (9:1; *v/v*); EtOH 70%: Ethanol-Water (70:30; *v/v*).

**Table 2:** Inhibitory effect (%) of *Ziziphus mauritiana* and *Balanites aegyptiaca* extracts on metabolic syndrome-related enzymes.

	Tested Enzymes		
Samples	α-Amylase	α-Glucosidase	Lipoxidase
*Z. mauritiana*	91.77 ± 2.00 ^a^	95.67 ± 0.38 ^a^	16.32 ± 0.99 ^a^
*B. aegyptiaca*	92.56 ± 2.02 ^a^	95.69 ± 0.14 ^a^	17.98 ± 1.07 ^a^

The means that share the same letter for each parameter show no significant difference at 0.05.

**Table 3:** The correlation (R^2^) between enzyme inhibition (%) and bioactive compounds and antioxidant power from *Ziziphus mauritiana* and *Balanites aegyptiaca*.

Tested Enzymes	TPC	TFC	TC	DPPH	ABTS
α-amylase	0.94	0.94	0.97	0.91	0.78
α-glucosidase	0.92	0.82	0.81	0.88	0.81
Lipoxidase	0.67	0.62	0.54	0.90	0.72

TPC: Total polyphenolic compounds; TFC: Total flavonoid compounds; TC: Total carotenoids; DPPH: 1.1-diphenyl-2-picrylhydrazyl; ABTS: 2.2 9-azinobis-3-ethylbenzothiazoline 6-sulfonic acid.

**Table 4:** Physicochemical parameters of microencapsulated *Ziziphus mauritiana* and *Balanites aegyptiaca extracts*.

Samples	Parameters				
	Encapsulation Yield (EY %)	Encapsulation Efficiency (EE %)	Moisture (%)	pH	Solubility (%)
*Z. mauritiana*	96.05	80.93 ± 1.71 ^a^	5.97 ± 0.65 ^b^	4.56 ± 0.01 ^b^	83.50 ± 0.44 ^a^
*B. aegyptiaca*	84.61	76.18 ± 1.39 ^b^	7.34 ± 0.32 ^a^	5.20 ± 0.03 ^a^	82.13 ± 0.15 ^b^

Different letters (a–b) for the same parameter and the same plant species show a significant difference between the means at 0.05.

**Table 5:** Bioactive compound content and antioxidant power of microencapsulated *Ziziphus mauritiana* and *Balanites aegyptiaca* extracts.

Parameters	*Z. mauritiana*	*B. aegyptiaca*
TPC (mg GAE/g DM)	20.75 ± 0.86 ^b^	23.09 ± 1.05 ^a^
TFC (mg QE/g DM)	15.91 ± 1.38 ^a^	13.22 ± 0.19 ^b^
TC (µg/g DM)	2.46 ± 0.00 ^b^	11.11 ± 0.02 ^a^
β-Carotene (µg/g DM)	1.77 ± 0.00 ^b^	8.83 ± 0.04 ^a^
Lycopene (µg/g DM)	0.92 ± 0.00 ^b^	4.41 ± 0.00 ^a^
DPPH (µM TE/g DM)	14.40 ± 1.66 ^a^	17.87 ± 1.77 ^a^
ABTS (µM TE/g DM)	86.81 ± 1.47 ^a^	74.41 ± 2.10 ^b^

TPC: Total polyphenolic compounds; TFC: Total flavonoid compounds; TC: Total carotenoids. Different letters (a–b) for the same parameter show a significant difference between the means of the plant species at 0.05.

**Table 6:** Bioactive compounds’ concentrations obtained by the liquid chromatographic analysis of *Ziziphus mauritiana* and *Balanites aegyptiaca* extracts.

Bioactive Compounds	Concentrations (mg/100 g DW Powder)			
	*Z. mauritiana*		*B. aegyptiaca*	
	280 nm	320 nm	280 nm	320 nm
Chlorogenic acid	15.96 ± 0.44 ^b^	33.99 ± 3.85 ^a^	33.99 ± 3.85 ^a^	26.69 ± 2.85 ^a^
(-)—Epicatechin	116.40 ± 4.09 ^a^	250.99 ± 3.72 ^a^	250.99 ± 3.72 ^a^	231.52 ± 5.06 ^a^
Caffeic acid	2.39 ± 0.30 ^a^	2.58 ± 0.85 ^a^	2.58 ± 0.85 ^a^	1.91 ± 0.19 ^a^
Gallic acid	2.72 ± 0.54 ^a^	ND	ND	ND
Quercetin	2.27 ± 0.06 ^a^	ND	ND	ND
Isorhamnetin	0.27 ± 0.00 ^a^	ND	ND	ND

ND—not determined. Different letters in a row denote significant differences between the bioactive compound concentrations for the same wavelength (*p* < 0.05).

## Data Availability

Not applicable.
